# Paternalism and autonomy: views of patients and providers in a transitional (post-communist) country

**DOI:** 10.1186/s12910-015-0059-z

**Published:** 2015-09-29

**Authors:** Lucija Murgic, Philip C. Hébert, Slavica Sovic, Gordana Pavlekovic

**Affiliations:** Department of Educational Technology, Andrija Stampar School of Public Health, School of Medicine, University of Zagreb, Zagreb, Croatia; Department of Family and Community Medicine, University of Toronto, Toronto, Canada; Department of Medical Statistics, Epidemiology and Medical Informatics, Andrija Stampar School of Public Health, School of Medicine, University of Zagreb, Zagreb, Croatia; Department for Social Medicine and Organization of Health Care, Andrija Stampar School of Public Health, School of Medicine, University of Zagreb, Zagreb, Croatia

**Keywords:** Patient autonomy, Ethics, Paternalism, Transitional country

## Abstract

**Background:**

Patient autonomy is a fundamental, yet challenging, principle of professional medical ethics. The idea that individual patients should have the freedom to make choices about their lives, including medical matters, has become increasingly prominent in current literature. However, this has not always been the case, especially in communist countries where paternalistic attitudes have been interwoven into all relationships including medical ones. Patients’ expectations and the role of the doctor in the patient-physician relationship are changing. Croatia, as a transitional country, is currently undergoing this particular process.

**Methods:**

Qualitative research was conducted by means of six focus group discussions held in the years 2012 and 2013 in Croatia. Focus groups were held separately with each of the following: first year and final (6^th^) year medical students, physicians engaged in medical ethics education, physicians practicing in a clinical hospital, family medicine residents and individuals representing patients with chronic disease. This research specifically addresses issues related to patient autonomy, in particular, the principles of truth telling, confidentiality, and informed consent. All focus group discussions were audio taped and then transcribed verbatim and systematized according to acknowledged qualitative analysis methods.

**Results and discussion:**

Patient autonomy is much more than a simple notion defined as the patient’s right to make treatment decisions independently. It has to be understood in context of the broader socio-cultural setting. At present, both patients and medical doctors in Croatia are increasingly appreciating the importance of promoting the principle of autonomy in medical decision-making. However, the current views of medical students, physicians and patients reveal inconsistencies.

**Conclusions:**

Knowing how to respect the various facets of patients’ autonomy should be part of physician’s professional duties, and also be reflected in his or her core clinical competencies. For this reason greater importance should be dedicated to patient autonomy issues in medical education in Croatia.

## Background

Patient autonomy is a fundamental principle of professional medical ethics. The ability to recognize and foster it, and its various dimensions, is widely considered an important clinical competency for physicians. However, its conception in the medical and ethical literature, as well as its practical implementation, still raises ongoing challenges for the practice of medicine.

Since the release of *The Principles of Biomedical Ethics* in 1977 by Beauchamp and Childress and its subsequent editions (now in its 7^th^ edition), autonomy has been widely accepted as one of the four principles of medical ethics together with the principles of beneficence, non-maleficence and justice [1] and has served as the philosophical underpinning for a variety of American reports and Presidential commissions of lasting import [2–4].

In the medical literature authors generally utilize this “liberal individualistic concept of autonomy” where, ideally, patients are decision makers who “act intentionally, with understanding, and without controlling influences, external and internal, that determine their actions” [5]. However, this influential view of patient autonomy has been criticized from many different perspectives [6, 7]. Particularly problematic in clinical practice is when autonomy is conflated with the notion of abstract self-concern or with solo decision-making [8]. Other concepts of patient autonomy are also described in the literature [5, 8–10].

On the spectrum of differing levels of independence, paternalism stands on the opposite side of autonomy. The objective of paternalism, like that of autonomy, is the good of the same moral agent, the patient [9]. Paternalism has been one of the traditional characteristics of the therapeutic relationship in medicine [9]. It implies that the physician makes decisions based on what he or she discerns to be in the patient’s best interests, even for those patients *who could make the decisions for themselves* [11]. This attitude presumes that physicians always know better than the patient what is good for the patient. It is precisely this representation of the physician-patient relationship that has suffered the harshest criticisms [9].

Yet, it is somewhat confusing when, in some jurisdictions like Croatia, on the one hand, medical paternalism appears to be trumped by autonomy, while on the other hand, many individual patients still expect, hope for, and even urge (in both subtle and outright ways) the doctor to be paternalistic [9]. It is understandably difficult for practicing physicians to deal with this attitudinal conflict.

The aim of this study was to compare and contrast views of different groups on patient autonomy issues in a post-communist, central European country with a strongly paternalistic background (Croatia separated from Yugoslavia in 1990, and a new democratic state was founded with the first democratically elected president and establishment of the first parliament in the same year).

It is argued that, in clinical practice in Croatia, more emphasis is still put on beneficence and physician-based decision-making rather than on patient autonomy.

## Methods

A qualitative research was conducted by means of six focus group discussions held in 2012 and 2013 in Croatia. The focus group method was chosen since it allows in-depth discussion and collection of opinions from more than one person in one session. Also, data generated through the interaction between group participants, results in a richer elaboration on a topic and a broader insight into understanding an issue than can be obtained from one-to-one interviews [12–14].

Focus groups were held separately with the following: first year medical students (MS1, 10 participants); final year medical students (MS6, 9 participants), all from the Zagreb School of Medicine; physicians engaged in medical ethics teaching at the Zagreb School of Medicine (ME, 8 participants), physicians practicing in one of the clinical hospitals in Zagreb, (MP, 11 participants), family medicine residents undergoing their training (FM, 9 participants); and participants representing patients with chronic disease (CP, 9 participants). Altogether, 56 voluntary participants took part in this study and all of them read and signed the provided consent form after the main researcher explained the research. All the students who were given the option to participate consented since the group was held during their medical ethics seminar class. Patients also were eager to share their experience. Many approached physicians, however, because of their conflicting schedules had to decline participation. Thus, forming a representative group was a significant challenge. Nevertheless, we formed homogeneous groups with a purposive sampling of a specific population of participants to minimize the influence of one opinion or perspective over others. Number, size, and group composition were formed to ensure participants felt comfortable and free to express their opinion [15]. Questions were prepared in advance, the same for all, but modified for the patient group.

A moderator (LM) conducted the first focus group (FM), led the discussion and took short notes. An assistant moderator (GP) also took notes and handled logistics. However, for the following focus groups it proved sufficient and practically more feasible to have one moderator (LM). No incentives were offered except for refreshments.

This research specifically addressed issues related to patient autonomy, that is, the central principles of truth telling, confidentiality and informed consent. Participants were asked to describe ethical problems encountered in their practice or experience and specifically describe how they addressed these problems, their reactions to them, and any additional comments regarding the situations. All audio taped materials were transcribed verbatim.

We analyzed our focus group interviews bearing in mind the research question we were examining; how the participants viewed different situations regarding patient autonomy issues. Themes were identified independently for each group and then merged together. All themes and comments were arranged with the word-processing method, which was performed by the main researcher (LM). Two additional researchers (GP and SS) helped in data interpretation by examining trends and patterns. Themes that emerged only in one or two groups were also considered. Since much prior research has been performed on patient autonomy issues, we used a directed approach to content analysis [16, 17]. Thus, using existing theory, we identified key concepts as initial coding categories [17]. Data that could not be initially coded was identified and analyzed later to determine if it represents a new category or a subcategory of an existing code.

Ethics approval for the study was sought and obtained from the University of Zagreb Medical School Research Ethics Committee.

## Results

As expected, all participants in the focus groups frequently encountered patient autonomy issues. Generally, these ethical concerns were mentioned in the context of their violation; however, examples of exemplary conduct were also presented. The relative importance of each of the observed dilemmas varied significantly among participants and was influenced by each participant’s own beliefs and values as well as life and professional experiences and age.

From the patients’ perspective, the most troublesome ethical problem as regards autonomy is the lack of privacy, especially in hospital settings. Most medical education in Croatia, as elsewhere, takes place in clinical settings, usually a hospital. Because there is often more than one patient in the room, it is hard to ensure privacy. Sometimes this is due to irresolvable organizational and practical problems such as: crowded rooms, lack of space dedicated for medical education and also lack of time. Too frequently, however, according to participants (CP), it is exacerbated either by neglect or by the ‘paternalistic mentality’ and authoritative approach of the responsible staff. They think that many physicians do not consider patient privacy to be an important issue.

During the discussion with other participants (MS1, MS6, MP, ME) it became obvious that students and practicing physicians are often unfamiliar with professional duties regarding patient privacy and how to ensure privacy in teaching hospitals. One of the students viewed patient privacy as problematic for learning:

*“I remember taking a history from a lady with my colleague. At one point her husband entered the room and literally expelled us out [from the room]. We went to our mentor who told us that it is our right and duty to speak to patients but still he didn’t oppose the husband. The thing is that we were not sure what are we supposed to do in such occasions”*. (MS6)

On the other hand, there were times when patients had little hope of privacy and no opportunity to express their wishes; which seemed disrespectful and outdated to some participants. One of the experienced clinicians recalls:

*“When I was a student… I remember several very unpleasant situations with patients… 15 of us packed in a clinic watching the patient and no one asked the patient if he was accepting [of] it. It was implied that if you came to a teaching hospital students would be there…”* (ME)

Recently, other problems have appeared. Modern information technologies pose challenges to privacy and confidentiality [18]. Great effort has been undertaken to protect data (personal health information), but this protection is not consistently applied on all levels. For example, to obtain coverage from the national insurance fund, patient-identifying data, such as their names and diagnoses, have to be sent to a central office. There, even though the company and its employees have a duty to respect confidentiality, confidential medical information is still potentially accessible by unauthorized employees. According to hospital physicians, the electronic records of patients in hospital environments, as well as other written documents such as medical histories, are not adequately protected by privacy standards.

“*Regarding privacy and confidentiality issues… they have never been as violated as now with this electronic data system. All patient data is online. We only have to enter our name and password and we can reach the medical history of any patient“.* (MP)

Private providers keep family medicine electronic records and some family physicians are worried that the commitment to confidentiality may thus be questionable. Another problem, according to participants (MP, FM), is that patients’ medical records, often psychiatric, can be revealed for court purposes. Testimony is generally required from more than one expert and people not in the patient’s circle of care can readily obtain access to private health information.

Further findings suggest that patients, being raised in a paternalistic environment like Croatia’s, do not quickly adopt more robust attitudes regarding autonomy and privacy. For example, people come to a doctor’s office or they telephone to enquire about the health of their spouse, another member of the family or sometimes even their neighbor. Employers also sometimes call to ask about their employee’s health. Such inquiries are seen as problematic even though they are frequently tolerated.

*“I had a patient who demanded from us to keep in secret the fact that he is in our hospital. And I had great difficulties to keep that, just technically speaking… because in our community and mentality it is normal that the doorkeeper has the list of all the names and directs the visitors. We presume that people want visits… but I think that better emphasis should be put on [the] patient’s preference”.* (ME)

It is often a custom that various medical certificates (e.g. a sick leave certificate, a medical certificate required in order to apply for a job or educational facility) have diagnoses encrypted in ICD-10^1^ on them. This is not really an encryption because employers or other parties involved can readily look for the proper meaning of these diagnoses on the Internet. Family practitioners were especially concerned about this problem since they issue these certificates.

“*It really annoys me that the system requires us to write precise diagnoses on the sick leave certificate. Why should the employer have to know which particular disease his employee has? If I, as a professional, state that a sick leave is required, that should be enough”.* (FM)

Practical problems were also identified in distinguishing between legal requirements and ethical concerns when writing patients’ diagnoses related to milder neurologic or psychiatric disorders on medical certificates required for a driving license or a work permit. Sometimes purposefully omitting a diagnosis seemed ethically more appropriate. One family physician admitted:

*“Once, a young nurse came to me. She requested documentation of all her past history in order to apply for a job. I found a record of psychiatric treatment from 10 years ago but after that she was fine. I didn’t mention that in the certificate since that could easily reduce her chances of getting a job. On balance, I decided not to record it”.* (FM)

Professionally however, the most troublesome ethical problems arose over the patient’s right to information and truth telling: what to tell, how to tell, when to tell, and whom to tell the patient’s diagnosis and prognosis.

### What to tell

A discussion regarding disclosure of a diagnosis arose in all focus groups and addressed two main issues: what kind of information should be provided to patients (its ‘qualitative content’) and how much information should be provided (its ‘quantitative extent’).

Regarding the qualitative content of the information, several viewpoints were raised. As one of the participants vividly described (MP), truth in a medical environment is a very thin and malleable concept. Nevertheless, all could agree that in theory one has to be sincere and tell the truth. For some participants, however, the truth can be packaged differently.

*“Telling the truth is not the only criterion of ethical behavior. Ethical norms should certainly be respected, but it is an art to know when something is possible to tell or not. You need a feeling for that. One should never lie, but if the truth is too difficult for the patient to bear you have to put it in a certain frame”*. (ME)

*“Nobody should lie. That’s true, but to lie is not the same as not to tell something. This is a very fine distinction”.* (ME)

This could be interpreted as a defense of deception; however, many doctors in our study saw it as a question of professional discretion, of so-called ‘therapeutic privilege’. Some of them admit to having lied sometimes purposefully, or at least omitted the truth, justifying it by claiming that it had a positive effect on their patients. They implied that less than the full truth can lead to a better medical outcome -- this was defended vigorously by some as the cases “ended well”.

*“From my experience, there are people who are just not ready to accept the truth about their diagnosis, regardless of their cognitive abilities and intellectual status. I admit that, at the beginning of my medical career I have sometimes asked the pathologists to write their findings differently and I can tell you that these cases ended well. Even in cases with highly educated patients involved.”* (ME)

The patients’ group, while strongly supporting truth telling (recognizing it to be in their best interest and especially important to protect their dignity), acknowledged that not all patients could endure the truth. Physicians, they said, have to be skilled, educated and aware of how to disclose the truth. One participant from the patient group explained it vividly:

*“I agree with all you [other participants] said about the importance of us [patients] knowing what’ s actually going on with us… but I wonder sometimes, if all this information we hear confuses us and burdens us more that we can bear.”* (CP)

However, the disclosure of bad news can be an especially stressful experience for family practitioners because of their close connection with patients. This makes them prone to emotional decision-making and consequently also prone to paternalism. One emotionally understandable but ethically questionable approach was described as follows:

*“When it is possible I try to exempt my patients from the truth. But I try to speak to the patients’ family. I think that it is very important, also from a practical point of view. Family members have to be prepared for all. They will have to care for him*.” (FM)

Regarding the quantitative extent of the information provided, two approaches were found as regards revealing the diagnosis to the patient. The first is an’opting out’ approach where the doctor presumes the patient wants to know the diagnosis unless the patient tells him openly and directly he doesn’t want to hear it.

*“We, as certified medical professionals, not only should be able to but are obliged to evaluate how much someone is prepared and accountable to hear his diagnosis. The patient has an indisputable right to know his diagnosis unless he tells us he doesn’t want to hear it, which happens in my practice extremely rarely. The patient needs to know what to expect. That is our job”*. (MP)

The second approach to truth telling is ‘opting-in’: the physician urges the patient to ask him what he wants to know and the physician then slowly unwinds the story until the patient is satisfied. As one of the experienced physician simply described it:

*“I have a tactic, I always ask the patient – what do you think your problem is? Do you have any questions for me?”* (ME)

One concern, frequently alluded to by our physician participants (MP, FM), was that the true diagnosis (if unexpected or very poor) shouldn’t be revealed to the patient if such disclosure would threaten the patient’s already frail medical condition. This paternalistic attitude was countered by some participants (MP) who promptly agreed with a participating cardiologist who stated that:

“*From evidence based physiological point of view, not to tell a patient that he suffered a heart attack because of fearing that it would make him worse, has little foundation*”. (MP)

Nevertheless, it is important to note here that the literature reveals preferences of patients to generally be informed [19, 20]. Albeit information disclosure can have harmful effects on patients [21, 22] it is now usually thought that benefits of disclosure greatly outweigh potential harms [18].

### How to tell

According to our findings the age of the patient and his cognitive abilities play an important role in the way and amount of information that can be given to him. Also, what is important is the terminology used. As one doctor explained:

*“My grandmother had lung cancer; however, I never told her she had a cancer but a tumor. For her tumor is ok even though she knows it is a serious illness. But the word ‘cancer’ would devastate her. So, we just used the word tumor instead”.* (MP)

Patients in our study felt the same and greatly appreciated that way of communicating. They frequently more readily accepted the term *tumor* rather than that of *cancer*.

Medical students were particularly critical of the lack of communication in hospital settings. They were greatly distressed by seeing and talking to patients lying in hospital beds who wanted to know the diagnosis but didn’t have chance to speak to the doctors or the doctors were ‘hiding’ behind technical terminology. On the other hand, students were startled, as were patients, when a difficult diagnosis was revealed too openly – that is, callously straightforward - which they perceived as being crude and thoughtless.

*“During our internal medicine rounds I remember meeting many patients not aware of their diagnosis. I would frequently question myself why is it that they are so oblivious? Equally awkward was witnessing a thoughtlessly straightforward announcement of poor prognosis to such a patient”.* (MS6)

### When to tell

The timing of the disclosure of a diagnosis and prognosis is very important. Generally, all our participants could agree that it should be soon, although not before the patient is ready to hear it. Yet, it is not easy to judge when circumstances are right. Particular concerns over patients with psychiatric illness were expressed.

*“What does it mean to be forthright and immediately earnestly reveal a diagnosis to a psychiatric patient? The right timing here is very important. For a lawyer, for someone who has not seen the patient it is all the same - they would just tell it and it’s done. But for us who see them daily it is not the same”.* (MP)

On the other hand, the patient group was concerned regarding the delayed diagnosis of a condition with a poor prognosis.

*“The worst sensation of my lying in a hospital bed was the ‘conspiracy of silence’ of the hospital staff. I was lying there for days feeling really sick and miserable. I was undergoing some diagnostic procedures but no one would tell me what they were suspecting. When I asked them they would only pronounce some, to me, not understandable medical jargon or say they didn’t know anything yet”.* (CP)

### Whom to tell

It has been generally acknowledged that the patient is the one who should be told first about his condition and it is his or her choice whom to tell later. In practice, in Croatia, in the hospital setting as well as in family practice, there are frequent requests from family members not to reveal the diagnosis to the patient. This fact raises moral concerns for the doctors in charge. Such requests are usually justified with an archaic explanation that those patients should not be bothered with these problems, even more so because they tend to be older patients and it is generally accepted that family members know best what is in the patient’s best interests.

However, there are examples of appropriate ways of dealing with such situations. An experienced and sensitive clinician described his style and methods of dealing with such situations:

*“I work with critically ill and challenging patients. When their family comes I make them sit down and I go to ask the patient – can I speak to your family about your condition? And the majority, 99 % says yes, of course”.* (ME)

Regarding informed consent, it is widely accepted that, for the patient who seeks a physician’s help, his or her consent to treatment is implied. Written consent forms were introduced in Croatian hospitals only a decade ago. They have been primarily used to address legal concerns (to protect the physician and the hospital) and are still frequently administered to patients without first providing sufficient information. Even though great care has been taken in writing some consent forms, patients complain that they often do not understand the terminology. Some students observed that the forms are often administered by nurses or by students themselves who may not appreciate all that is involved in the procedure. Thus, consent becomes a formulaic process and patients largely accept it. An experienced specialist stated:

*“If I were the patient I would ask them to explain everything to me… if it would hurt me or not? What is the risk? And so forth… but the majority of my patients trust me and they just say – whatever you say, Mr. Doctor. Not all of them say it in such an explicit way but they mean it. Our duty should be to ask them whether they have some more questions. If everything is clear they can sign it. But they don’t like that; they just say I will sign whatever you say is needed”*. (ME)

However, patients are sometimes so overwhelmed with the hospital setting or the news that they don’t know what to ask, especially if they are not encouraged by the health care professionals to ask questions or to raise concerns.

### Autonomy vs. beneficence

In situations where a diagnosis is required to be disclosed to the authorities a ‘paternalistic approach’ is readily accepted by many focus group participants (MS1, MS6, MP, FM, ME). The perception is that there is a need to advance a larger social good -- for example, to protect the community from patients with communicable diseases or from psychiatric patients who are a danger to others. One participant observed the negative side of this attitude.

*“We are used to caring for the community, or better yet, for the collective good and not as aware of the problems of a particular individual. I think that now we have to slowly change that and give individual autonomy a higher [place in the] hierarchy of values than before.”* (ME)

This statement was articulated during a very emotional discussion that arose (in the ME group) regarding the duty of a person to disclose his HIV positive status to the spouse/partner. The majority of participants (the same was in MS1, MS6 and FM groups) were more concerned about protecting the spouse or other close contacts. By contrast, practitioners with more experience in treating such patients had different views - they were more concerned for the patient himself. This focus on the patient’s interests can also have a positive public health benefit in terms of increased patient trust in the medical system.

*“It is good for the patient to have one doctor to whom he can trust because that doctor protects his privacy. I can tell you that we would have a bigger public health problem if these people wouldn’t trust the health system and wouldn’t come to be treated. Another very important thing is to talk to them, to communicate and to find out the circumstances in which they live”.* (ME)

### Medical ethics education

It is worth mentioning that some medical students and residents learn (at an early point in their education) that deception can be an acceptable option. For example, when they are given the opportunity to do something practical, informed consent is often not sought from the patient -- or, if the students are introduced to the patients -- they are often called ‘young doctors’.

*“I was on the cardiac surgery round and during the operation I was given the possibility to sew up (suture) the lower leg from where the vein graft was harvested. I surely transgressed the principle of informed consent because no one had informed the patient that a student would sew him, but at that time I didn’t even think about it.”* (MS6)

Participants from groups consisting of medical students, family physicians and hospital clinicians complained that medical education, including residency programs, failed to instruct students and residents how to deal with the frequent ethical dilemmas that arise regarding patient autonomy. The only ethical issues they remembered were debates on the right to abortion or euthanasia. More mundane ethical issues just didn’t appear to be important enough to be highlighted during their medical education. The discourse on patients’ autonomy during medical education was focused on the legal liability of physicians and how to protect themselves from a law suit, rather than how to behave ethically. Students (MS1, MS6) admitted that they were greatly influenced by their teachers as role models. Guidelines and algorithms on how to behave ethically would be welcomed.

## Discussion

The results of our study confirmed that ethical dilemmas regarding patient autonomy – so thickly described in medical literature [23–25] -- appear frequently in medical practice in Croatia and that there, as in other countries, physicians often find themselves unprepared to tackle them [26–28]. As described in literature too, a range of approaches could be found in dealing with these issues: from the strictly paternalistic [29] where the physician is the one who decides what is best for patients to those more libertarian in orientation which emphasize patient self-determination as the ultimate principle [30–32]. However, it is worth emphasizing that physicians in Croatia should not generally be considered paternalistic since their approaches largely varied according to their individual attitudes as well as patient characteristics (age, cognitive status, and expectations) and the broader socio-cultural context. Our study suggests a broad range of concern regarding autonomy issues among Croatian patients, medical students, residents and practitioners but participants identified few ethical resources to help address these issues.

The idea that individual patients should have the freedom to make choices about their lives, thus medical matters, has become increasingly prominent in democratic countries over the past 50 years [18]. According to all our focus group participants, generally speaking, more emphasis is starting to be placed on patient autonomy issues in Croatian medical facilities today. For example, information is more likely to be provided to patients, patient consent is explicitly sought, and it is increasingly acknowledged that patients have the right to make medical decisions about themselves. However, despite these changes in medical practice, we found sufficient evidence that medical paternalism remains embedded in the medical profession [33].

The concept of autonomy, itself, can be differently interpreted [5, 6, 8, 10]. In the literature, the meaning of autonomy, the different types of autonomy [34], as well as the impairments of autonomy (for example, temporary or permanent), have been widely discussed. Our participants did not debate the philosophical meaning of autonomy nor were they asked to define what they meant by autonomy. Their understanding of autonomy is generally in line with liberal individualist ideas [1] and conforms to what they have been taught at medical school [35].

Some authors argue that in modern healthcare autonomy is considered an obligation equivalent to, or even more compelling than, the principle of beneficence [6, 8, 10]. Autonomy denotes providing care that “patients will find beneficial according to their own values and beliefs, care that also helps to empower them and maximize their opportunities for self-actualization” [18]. However, while this understanding of patient autonomy is held in high regard in places like the United States and Canada, in many cultures, for example in some Latin America [31] and Asian societies [32], a still significant number of physicians and families believe in paternalism as a form of beneficence [31]. In these societies it is usually not the physician and patient that form the core decision-making unit. It is the family or a family representative who plays a pivotal rule in decision making since the family’s autonomy and well-being is regarded as more important than the individual person’s autonomy and well-being. In such a cultural scenario, the illness and its management are commonly considered a shared responsibility of the family [31, 32].

Even though there is empirical evidence of a paternalistic mentality persisting in some post-communist countries [29], little research has been done in this field. We believe our study has added to the slender knowledge regarding the changing attitudes in transitional countries about patient autonomy issues. However, further research should be conducted in order to understand how these attitudes are related to the collapse of authoritarian regimes.

One point also worth clarifying here is the relevancy of the focus on privacy and confidentiality to the autonomy/paternalism question. The right to privacy, meaning the right to be free from intrusions or interference, is a fundamental tenet of liberal democracies and is critical to any working concept of personal autonomy [18]. Confidentiality refers to the duty of healthcare professionals to respect patient whishes regarding the collection, use and disclosure of their private information. Consequently, the management of personal health information is contentious because there is tension between several professional responsibilities; breaching the right to privacy threatens the trust of the patient in the healthcare system but, on the other hand, physicians cannot do an adequate job providing care if they are unable to adequately ‘invade’ a patient’s physical and mental privacy [18].

Truth is another element vital to advancing autonomy. However, opinions on truth telling, among our study participants, also varied. As has been noted by other researchers, some patients ‘wish to be deceived’ or at least not to be told ‘everything’ [18]. In particular, there is a distinct group of patients which desires all the information about the diagnosis but does not necessarily wish detailed information about the prognosis [36]. This conflict between the patient’s right to know and powerful cultural factors that militate against disclosure is one of the most difficult problems in the patient-physician relationship [31]. It was particularly acknowledged to be so by the clinicians (MP, FM, ME) in this study.

In Croatia as well as in other countries worldwide, patients often welcome paternalism, even authoritarianism, in medical issues. In other words, they voluntarily relinquish their autonomy in the name of ‘doctor-knows-best’ [9, 29, 31, 32]. Some of the explanations found in literature for this attitude are that patients lack confidence, are not sure which option they prefer, have conflicting priorities, or anticipate blaming themselves if outcomes are poor [7]. This is in contrast to notions from the literature that paternalism should simply be rejected [33]. Our study supports the idea that paternalism should be modernized, not rejected all together, but adapted to attitudes that cannot change overnight.

For many study participants, nevertheless, paternalism is not incompatible with the principle of autonomy, especially when it puts the patient’s good above all other considerations. According to this view, some form of paternalism is justified in many therapeutic relationships due to the nature of critical illness and the diminished role of the sick [9]. Illness itself causes a state of diminished autonomy or even a complete loss of autonomy. This allows paternalism, especially in countries such as Croatia, to fill the vacuum that is left [9, 37]. This is not necessarily a bad thing but requires more work and attention.

Here emerges the important role of medical education. From our research it seems that in Croatia, neither the written nor the ‘hidden curriculum’[38] sufficiently emphasize the importance of patient autonomy and its various aspects. Recognizing this deficiency, medical students, family practitioners and hospital physicians were all eager to have some practical guidelines or even algorithms to help them resolve conflicting situations.

However, despite deficiencies in education, the role of the physician in the patient-physician relationship is changing worldwide [39] and also in Croatia. Specifically, it has been shifting from paternalism to shared decision-making which is considered to be one of the characteristic features of patient centered care, a leading ideology of modern medicine and healthcare [10, 11]. For such a model to function effectively, the following elements are essential: the maintenance of trust in the physician-patient relationship; respect; and the adoption of patient-centered communication [31, 40]. The concept of good medicine has always been based on mutual trust [41] and its success rests as much in art and acting as in science and evidence [18].

This study’s conclusions are limited by its methodological deficiencies. It is a pilot study of the attitudes regarding autonomy issues in Croatia and it was conducted on a relatively small number of participants. Nevertheless, our opinion is that it contributes to a better understanding of autonomy issues in transitional countries. It is unique because it assesses focus groups participants from different perspectives in the healthcare system; patients themselves, first and final year medical students, hospital and family physicians, and medical ethics teachers.

In future research it will be important to investigate more deeply the influence of changing socio- political conditions on attitudes and actual medical practice regarding patients’ autonomy.

## Conclusion

It can be concluded that for Croatian physicians and patients, autonomy requires more than an allegiance to the simple notion of the patient’s right to make treatment decisions independently. Despite common inconsistencies in views, attitudes and behavior regarding patient autonomy issues and the constant collision with our paternalistic background, both patients and physicians in Croatia are beginning to appreciate more the importance of the principle of autonomy in medical decision-making. It is too early to tell whether Croatian physicians will continue in the direction of more individualized decision-making or whether paternalistic practices will predominate. Nevertheless, greater importance should be dedicated to patient autonomy issues in medical education.

## Abbreviations

MS1: First year medical students
MS6: Final year medical students
ME: Medical doctors engaged in medical ethics teaching
MP: Medical doctors practicing in a clinical hospital
FM: Family medicine residents
CP: Patients with chronic disease

## Footnotes

1 **ICD-10** is the 10th revision of the International Statistical Classification of Diseases and Related Health Problems (ICD), a medical classification list by the World Health Organization (WHO). It contains codes for diseases, signs and symptoms, abnormal findings, complaints, social circumstances, and external causes of injury or diseases.
